# Magnetic Resonance Imaging Interpretation in Patients with Sciatica Who Are Potential Candidates for Lumbar Disc Surgery

**DOI:** 10.1371/journal.pone.0068411

**Published:** 2013-07-10

**Authors:** Abdelilah el Barzouhi, Carmen L. A. M. Vleggeert-Lankamp, Geert J. Lycklama à Nijeholt, Bas F. Van der Kallen, Wilbert B. van den Hout, Annemieke J. H. Verwoerd, Bart W. Koes, Wilco C. Peul, for the Leiden–The Hague Spine Intervention Prognostic Study Group

**Affiliations:** 1 Department of Neurosurgery, Leiden University Medical Center, Leiden, The Netherlands; 2 Department of Radiology, Medical Center Haaglanden, the Hague, The Netherlands; 3 Department of Medical Decision Making, Leiden University Medical Center, Leiden, The Netherlands; 4 Department of General Practice, ErasmusMC, University Medical Center, Rotterdam, The Netherlands; 5 Department of Neurosurgery, Medical Center Haaglanden, the Hague, The Netherlands; Charité University Medicine Berlin, Germany

## Abstract

**Background:**

Magnetic Resonance Imaging (MRI) is considered the mainstay imaging investigation in patients suspected of lumbar disc herniations. Both imaging and clinical findings determine the final decision of surgery. The objective of this study was to assess MRI observer variation in patients with sciatica who are potential candidates for lumbar disc surgery.

**Methods:**

Patients for this study were potential candidates (n = 395) for lumbar disc surgery who underwent MRI to assess eligibility for a randomized trial. Two neuroradiologists and one neurosurgeon independently evaluated all MRIs. A four point scale was used for both probability of disc herniation and root compression, ranging from definitely present to definitely absent. Multiple characteristics of the degenerated disc herniation were scored. For inter-agreement analysis absolute agreements and kappa coefficients were used. Kappa coefficients were categorized as poor (<0.00), slight (0.00–0.20), fair (0.21–0.40), moderate (0.41–0.60), substantial (0.61–0.80) and excellent (0.81–1.00) agreement.

**Results:**

Excellent agreement was found on the affected disc level (kappa range 0.81–0.86) and the nerve root that most likely caused the sciatic symptoms (kappa range 0.86–0.89). Interobserver agreement was moderate to substantial for the probability of disc herniation (kappa range 0.57–0.77) and the probability of nerve root compression (kappa range 0.42–0.69). Absolute pairwise agreement among the readers ranged from 90–94% regarding the question whether the probability of disc herniation on MRI was above or below 50%. Generally, moderate agreement was observed regarding the characteristics of the symptomatic disc level and of the herniated disc.

**Conclusion:**

The observer variation of MRI interpretation in potential candidates for lumbar disc surgery is satisfactory regarding characteristics most important in decision for surgery. However, there is considerable variation between observers in specific characteristics of the symptomatic disc level and herniated disc.

## Introduction

Sciatica is defined as intense leg pain in an area served by one or more spinal nerve roots and is occasionally accompanied by neurological deficit [1]. Sciatica places a heavy burden on public health as it is a major source of lost productivity [2]. The most common cause of sciatica is a herniated disc [1]. Magnetic resonance imaging (MRI) is considered the imaging procedure of choice for patients suspected of lumbar herniated discs [3], [4], [5]. MRI is indicated in patients with severe symptoms who fail to respond to conservative care for at least 6 to 8 weeks [1]. In these cases surgery as a treatment modality might be considered and MRI is used to assess if a herniated disc with nerve root compression is indeed present. Both imaging and clinical findings determine the final decision of surgery [6]. The important role of MRI in clinical decision making makes a reliable interpretation of lumbar MRI therefore desirable.

Despite remarkable advancements in diagnostic imaging and surgical techniques the results after lumbar disc surgery do not seem to have improved during recent decades: depending upon the used outcome measure, the results of lumbar disc surgery are unsatisfactory in 10 to 40% of the patients [7], [8], [9]. It has been suggested that the poor outcomes following lumbar disc surgery may be more often due to the errors in diagnosis than the surgical technique or its complications [6], [10]. For example, a false-positive diagnosis of nerve root compression on MRI may lead to unwarranted surgery. Therefore, if truly substantial interpretation variability exists among those who routinely interpret spine MRI studies, this would influence treatment decisions with possible negative effects. Unreliable interpretation may also pose research problems when attempting to uncover the relationship between specific imaging characteristics and patient outcomes. Therefore, insight in the interpretation variability of MRI findings among potential candidates for lumbar disc surgery is essential.

The investigators previously reported the results of a randomized controlled trial comparing early surgery with prolonged conservative care for patients with sciatica over one year’s follow-up [11]. The randomized patients were part of a larger group that underwent MRI to assess the eligibility for the trial. Within this larger group, we report on the intra- and inter-observer variation in MRI evaluation among two neuroradiologists and one neurosurgeon.

## Materials and Methods

### Ethics Statement

The medical ethics committees at the nine participating hospitals (Leiden University Medical Center, Medical Center Haaglanden, Diaconessen Hospital, Groene Hart Hospital, Reinier de Graaf Hospital, Spaarne Hospital, Bronovo Hospital, Rijnland Hospital and Lange Land Hospital) approved the protocol. Written informed consent was obtained from all patients.

### Study Population

Patients for this study were patients with 6 to 12 weeks of sciatic symptoms being so severe that they were eligible for surgery according to their family practitioners and were therefore referred to a neurologist. The attending neurologist subsequently evaluated whether these patients were eligible to participate in the Sciatica Trial: a multicenter randomized controlled trial designed to determine whether early surgery results in a more effective outcome compared to a strategy of prolonged conservative treatment with surgery if needed. Patients were excluded if they were presenting with cauda equina syndrome, insufficient strength to move against gravity, identical complaints in the previous 12 months, previous spine surgery, pregnancy, severe coexisting disease or if they were not between 18 to 65 years of age. All participants who were not meeting one or more of the aforementioned exclusion criteria underwent MRI. If the MRI showed a disc herniation with nerve root compression correlating with clinical symptoms according to the attending neurologist and neurosurgeon the corresponding patient was eligible to participate in the randomized clinical trial. Thus if a patient did not display a disc herniation according to the neurologist who assessed the MRI at the time of enrollment in the Trial, this patient could not enter the randomized controlled Trial. As the purpose of the current study was to evaluate observer variation among sciatica patients who are surgical candidates for sciatica, MRIs of all patients (regardless of participation in the randomized clinical trial) were again evaluated by independent observers (who did not participate in this study before) to determine observer variation regarding MRI characteristics. Details of the design and study protocol have been published previously [12].

### MRI Protocol and Image Evaluation

MRI scans were performed in all 9 participating hospitals using standardized protocols tailored to a 1.5 Tesla scanner. Sagittal T1 and axial T1 spin echo images of the lumbar spine were acquired. In addition, T2 weighted sagittal and axial series were obtained. For research purposes also contrast-enhanced (Gadolinium dithylene triamine penta-acetic acid [DTPA] at a standard dose of 0.1 mmol/kg body weight) T1 fat suppressed sagittal and axial images were obtained.

MR images of all included patients were obtained and saved in an Apple PowerBook PC laptop with an 1.67 GHz G4 processor running open-source OsiriX Medical Image software (Version 3.0.1). Size of the monitor was 15,2 inch, 1280×854 pixel resolution.

Two neuroradiologists and one neurosurgeon independently evaluated all MR images, blinded to clinical information. None of the readers had been involved in either the selection or care of the included patients. The readers were able to freely adjust contrast and image brightness and zoom, and were able to compare sagittal and axial images simultaneously. All readings were performed on the same Apple PC laptop. Observer experience in reading spine MRI’s was 7 and 6 years post-residency for the neuroradiologists and 4 years post-residency for the neurosurgeon.

Each reader received a manual containing definitions of imaging characteristics based on the recommendations from the combined task forces of the North American Spine Society, the American Society of Spine Radiology, and the American Society of Neuroradiology for classification of lumbar disc pathology in order to standardize the nomenclature [13]. Pictorial examples were also provided where appropriate, gathered from the literature if available. Vertebral endplate signal changes were defined according to criteria of Modic et al. [14], [15]. Before beginning the study, the readers met in person to review and refine the standardized definitions in case of ambiguities. After reaching final consensus, standardized case record forms with these final definitions were used to evaluate the images (Table 1). First, all readers had to choose whether the MRI showed an impaired lumbar disc level that may have explained the sciatic complaints of the patients. If so, multiple characteristics of the degenerated disc level and disc herniation were scored. For both the presence of disc herniation and nerve root compression a four point scale was used: “Definite about the presence”, “Probable about the presence” if there was some doubt but probability >50%, “Possible about the presence” if there was reason to consider but probability <50%, and “Definite about the absence”.

**Table 1:** MRI study variables.

MRI variable	Type	Categories
Disc level that most likely caused the lumbosacral radicular syndrome of the patient	Disc level	1. L2L3 2. L3L4 3. L4L5 4. L5S1 5. Not applicable, all disc levels have a normal disc contour: no disc extension beyond the normal margins of the intervertebral disc space at any disc level
	Disc contour at this disc level	1. Bulging: presence of disc tissue circumferentially (50–100%) beyond the edges of the ring apophyses 2. herniation: localized displacement of disc material beyond the normal margins of the intervertebral disc space
	Certainty about the presence ofthis disc herniation	1. Definite about the presence: no doubt about the presence 2. Probable about the presence: some doubt but likelihood >50% 3. Possible about the presence: reason to consider but likelihood <50% 4. Definite about the absence: no doubt about the absence
	Loss of disc height (distance between the planes of the end-plates of the vertebrae craniad and caudad to the disc) at this disc level	1. Yes 2. No
	Signal intensity of nucleus pulposus on T2 images at this level	1. Hypointensity 2. Normal 3. Hyperintensity
	Vertebral endplate signal changes upper endplate	1. No VESC 2. VESC type I: hypointense in T1-weighted sequences and hyperintense in T2-weighted sequences 3. VESC type II: hyperintense both in T1- and T2-weighted sequences 4. VESC type III: hypointense both in T1- and T2-weighted sequences 5. Mixed VESC type I/II 6. Mixed VESC type II/III
	Vertebral endplate signal changes lower endplate	1. No VESC 2. VESC type I 3. VESC type II 4. VESC type III 5. Mixed VESC type I/II 6. Mixed VESC type I/III
	Spinal canal stenosis	1. Yes 2. No
	Absence of epidural fat adjacentto the dural sac or surroundingthe nerve root sheath	1. Yes, completely disappeared 2. Yes, partly disappeared3. No disappearance
	Place of absence of epidural fat adjacent to the dural sac or surrounding the nerve root sheath	1. Sub-articular zone: zone, within the vertebral canal, sagittally between the plane of the medial edges of the pedicles and the plane of the medial edges of the facets, and coronally between the planes of the posterior surfaces of the vertebral bodies and the under anterior surfaces of the superior facets 2. Foraminal zone: zone between planes passing through the medial and lateral edges of the pedicles 3. Extra-foraminal zone: the zone beyond the sagittal plane of the lateral edges of the pedicles, having no well-defined lateral border
	Presence of impaired discs onother disc levels	1. Yes: presence of disc extension(s) beyond the normal margins of the intervertebral disc space at other disc levels 2. No: absence of disc extension(s) beyond the normal margins of the intervertebral disc space at other disc levels
If a herniation at the disc level is considered	Side of this disc herniation	1. Right 2. Left 3. Right and left
	Location on axial view of this disc herniation	1. Central zone: zone within the vertebral canal between sagittal planes through the medial edges of each facet 2. Sub-articular zone: zone, within the vertebral canal, sagittally between the plane of the medial edges of the pedicles and the plane of the medial edges of the facets, and coronally between the planes of the posterior surfaces of the vertebral bodies and the under anterior surfaces of the superior facets 3. Foraminal zone: zone between planes passing through the medial and lateral edges of the pedicles 4. Extra-foraminal zone: the zone beyond the sagittal plane of the lateral edges of the pedicles, having no well-defined lateral border
	Location on sagittal view ofthis disc herniation	1. Disc level: herniated disc between the end-plates of the vertebrae craniad and caudad to the disc 2. Folded upwards: disc tissue beyond the end-plate of the vertebrae craniad to the disc 3. Folded downwards: disc tissue beyond the end-plate of the vertebrae caudad to the disc
	Size of this disc herniation inrelation to spinal canal	1. Large ***stenosing: size >75%*** of the spinal canal ***2. Large: size 75–50%*** of the spinal canal 3. Average: size 25–50% of the spinal canal 4. Small: size <25% of the spinal canal
	Morphology	1. Protrusion: localized displacement of disc material beyond the intervertebral disc space, with the base against the disc of origin broader than any other imension of the protrusion 2. Extrusion: localized displacement of disc material beyond the intervertebral disc space, with the base agains the disc of origin narrower than any one distance between the edges of the disc material beyond the disc space measured in the same plane, or when no continuity exists between the disc material beyond the disc space and that within the disc space
Nerve root compression	Probability of nerve rootcompression	1. Definite about the presence: no doubt about the presence 2. Probable about the presence: some doubt but likelihood >50% 3. Possible about the presence: reason to consider but likelihood <50% 4. Definitely no nerve root compression
	If nerve root compression present, which nerve root is affected	1. L3 2. L4 3. L5 4. S1 5. Not applicable, definitely no nerve root compression
	Side nerve root compression	1. Right 2. Left
	Nerve root thickness distal to the site of compression	1. Normal 2. Thickened 3. Narrowed
	Flattening of the ventrolateral angle of the dural sac or the emerging root sheath	1. Yes 2. No

When all three observers finished reading the images they repeated the MRI evaluation for ten percent of the evaluated images to provide intra-observer reliability data. The observers were not aware they were actually evaluating the images for a second time since in advance they were not informed about the conduction of an intra-observer reliability study. The images used for this intra-observer study were randomly selected from the first three-quarter of the evaluated images to minimize possible effects of recent memories. The time period between the first and the second evaluation was at least 2 months for all observers.

### Statistical Analysis

To assess the intra- and inter-observer reliability, we used percentages of absolute agreement and kappa coefficients. Percentage of absolute agreement equals the number of cases for which the observers fully agree, proportional to the total number of cases [16]. A common interpretation of good agreement is 80% [17]. However, the absolute percentage of agreement is inadequate, because it does not discriminate between actual agreement and agreement which arises due to chance [18]. A measure which attempts to correct for this is the kappa statistic [19]. In case of ordered data, we calculated weighted kappa scores which is based on the idea that in any ordered scale some possible disagreements are more serious than others.

The kappa statistic is affected by the prevalence of the events [20], [21]. so that findings with very high or low prevalence lead to very low kappa values, even if the observer agreement is high [22]. Therefore, for both the intra- and inter-observer reliability we only calculated kappa values for findings reported in more than 10% and less than 90% of all reports [23].

Both weighted and unweighted kappa statistics were computed for all possible pairings of observers. In addition we computed overall unweighted kappa coefficients for multiple raters. When the number of raters is two, the kappa statistic is based on the observed proportion of agreement and the expected proportion of agreement. When there are more than 2 raters, STATA (the program used for all analyses, version 12,0) implemented formulas in its statistical package that can be found in the statistical book of Fleiss and co-authors [24]. While no absolute definitions have been accepted for the interpretation of kappa values, we used guidelines proposed by Landis and Koch for interpretation [25]. Values of less than 0.00 indicated poor; 0.00–0.20 slight; 0.21–0.40 fair; 0.41–0.60 moderate; 0.61–0.80 substantial; and 0.81–1.00 excellent or almost perfect agreement. Value of 0.21–0.60 indicates fair to moderate agreement and a value of 0.41–0.80 indicates moderate to substantial agreement.

In a subanalysis we calculated interobserver agreement when the probability of disc herniation or nerve root compression were dichotomized into “probability>50%” on one hand and “probability <50%” on the other hand. In a subanalysis we also calculated interobserver agreement in the patients who were not randomized.

## Results

**Figure 1:**
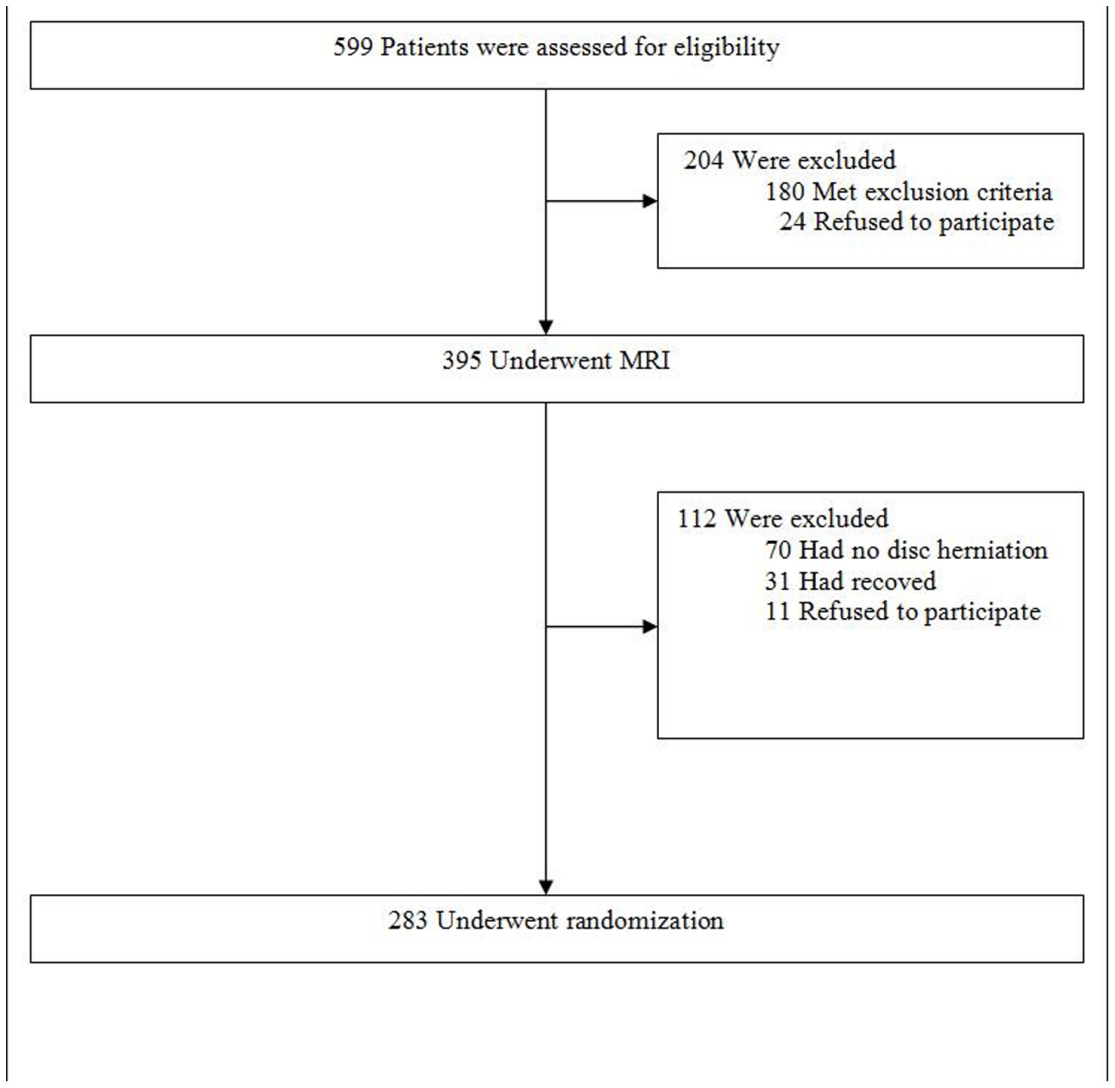
Flowchart.

Of the 599 patients screened for the study, 395 patients considered eligible for inclusion underwent MRI of whom 283 patients were randomized and 112 not (Figure 1). Reasons why 112 patients were not randomized was that 70 (63%) did not have a disc herniation according to the neurologist who assessed the MRI in one of the 9 participating centers at the time of enrollment (a visible disc herniation on MRI was a prerequisite to enter the Trial), 31 (28%) patients recovered before the randomization procedure could take place, and 11 (10%) patients refused to be randomized. In total, 283 baseline MRIs of the 283 randomized patients and 106 MRIs of the 112 non-randomized patients could be retrieved, bringing the total to 389 MRIs for the interagreement analysis between the MRI observers of the present study (2 neuroradiologists and one neurosurgeon, all 3 observers did not have participated in the study before).

The study population had a mean age of 43.2 years with the majority being men (63%). Of the 389 MRIs, there was a definite or probable disc herniation present in 87% of the MRIs according to reader A, in 84% according to reader B and in 79% according to reader C (neurosurgeon) (Table 2).

**Table 2:** Summary of the interpretation of 389 MRI images.

	Reader A	Reader B	Reader C
**Probability of disc herniation**			
*Definite*: no doubt about the presence of disc herniation	299 (76.9)	298 (76.6)	240 (61.7)
*Probable*: some doubt but probability >50%	38 (9.8)	28 (7.2)	67 (17.2)
*Possible*: reason to consider, but probability <50%	8 (2.1)	4 (1.0)	16 (4.1)
*Definitely no* disc herniation present	44 (11.3)	59 (15.2)	66 (17.0)
**Probability of nerve root compression**			
*Definite*: no doubt about the presence of nerve root compression	222 (57.1)	277 (71.2)	144 (37.0)
*Probable*: some doubt but likelihood >50%	97 (24.9)	43 (11.1)	120 (30.8)
*Possible*: reason to consider, but likelihood <50%	42 (10.8)	32 (8.2)	64 (16.5)
*Definitely no* nerve root compression present	28 (7.2)	37 (9.5)	61 (15.7)

Reader A en B represent the two neuroradiologists, while reader C represents the neurosurgeon.

Values are n (%).

The interobserver agreement was excellent for the disc level that was assumed to cause the sciatic symptoms of the patient (Table 3). Excellent agreement was also found on the question which nerve root was affected most. With use of a four point scale, interobserver agreement was moderate to substantial for the probability of disc herniation (kappa range 0.57–0.77). When dichotomizing the answers into “probability of disc herniation >50%” on one hand and “probability of disc herniation <50%” on the other hand, interobserver agreement was substantial (kappa range 0.67–0.75). With this dichotomized scale all three observers agreed in 88% of the MRIs whether the probability of disc herniation was above or below 50%. With use of a four point scale, interobserver agreement regarding the probability of nerve root compression was moderate to substantial (kappa range 0.42–0.69). In 50 percent of the evaluated MRIs the three observers disagreed on the probability of nerve root compression. The greatest source of reader discrepancy was between the category “definite about the presence” and “probable about the presence”, accounting for 58% of all disagreements across all reading pairs. When dichotomizing the answers into “probability of nerve root compression >50%” on one hand and “probability of nerve root compression <50%” on the other hand, interobserver agreement among the three readers was substantial (kappa range 0.60–0.80). With this dichotomized scale all three observers agreed in 82% of the MRIs whether the probability of nerve root compression was above or below 50%. In the subgroup consisting of patients who were not randomized, interobserver agreement regarding the probability of nerve root compression was lower than in the total group (Table 4). When dichotomizing the answers into “probability of nerve root compression >50%” and “probability of nerve root compression <50%” interobserver agreement was moderate to substantial (kappa range 0.45–0.69). Agreement between the neuroradiologists was higher compared to the agreement between the neurosurgeon and the neuroradiologists.

**Table 3:** Agreement among the readers.

	A vs B		A vs C		B vs C		All observers	
	%agreement	kappa	%agreement	kappa	%agreement	kappa	%agreement	multiraterkappa
Disc level that is assumed to cause the lumbosacral radicular syndrome ¶	92.0	0.86	88.4	0.81	90.5	0.84	86.4	0.84
Most affected nerve root (including side)	91.0	0.89	88.7	0.86	89.7	0.88	86.1	0.88
Probability of disc herniation (4 categories)∫	88.2	0.77	78.7	0.67	75.6	0.61	72.8	0.57
Probability of disc herniation (2 categories)‡	93.6	0.75	91.8	0.71	90.0	0.67	87.7	0.71
Probability of nerve root compression (4 categories)∫	75.1	0.69	59.9	0.56	57.1	0.51	49.9	0.42
Probability of nerve root compression (2 categories)‡	94.1	0.80	85.4	0.62	84.6	0.60	82.0	0.66

A en B represent the two neuroradiologists, while C represents the neurosurgeon. Analysis with the total number of patients (n = 389).

¶ The 5 categories were: 1) L2L3 2) L3L4 3) L4L5 4) L5S1 5) Not applicable, all disc levels have a normal disc contour (no disc extension beyond the normal margins of the intervertebral disc space at any lumbar disc level).

∫ The 4 categories were: 1) “Definite about the presence” if there was no doubt about the presence.

2) “Probable about the presence” if there was some doubt but the probability was >50%.

3) “Possible about the presence” if there was reason to consider but the probability was <50%, and 4) “Definite about the absence” if there was no doubt about the absence.

‡ The categories “Definite and probable about the presence” were combined to one category and the categories “possible about the presence” and “definite about the absence” were also combined to one category.

**Table 4:** Agreement among the readers.

	A vs B		A vs C		B vs C		All observers	
	%agreement	kappa	%agreement	kappa	%agreement	kappa	%agreement	multiraterkappa
Disc level that is assumed to cause the lumbosacral radicular syndrome ¶	78.3	0.68	61.3	0.47	70.8	0.59	58.5	0.57
Most affected nerve root (including side)	72.6	0.67	66.0	0.58	69.8	0.61	59.4	0.62
Probability of disc herniation (4 categories)∫	81.1	0.77	69.8	0.61	73.6	0.63	66.0	0.58
Probability of disc herniation (2 categories)‡	87.7	0.75	78.3	0.59	81.1	0.64	73.6	0.65
Probability of nerve root compression (4 categories)∫	61.3	0.65	42.5	0.43	48.1	0.42	36.8	0.32
Probability of nerve root compression (2 categories)‡	84.9	0.69	72.6	0.48	70.8	0.45	64.2	0.52

A en B represent the two neuroradiologists, while C represents the neurosurgeon. Sub analysis of the patients who did not undergo randomization (n = 106).

¶ The 5 categories were: 1) L2L3 2) L3L4 3) L4L5 4) L5S1 5) Not applicable, all disc levels have a normal disc contour: no disc extension beyond the normal margins of the intervertebral disc space at any disc level.

∫ The 4 categories were: 1) “Definite about the presence” if there was no doubt about the presence.

2) “Probable about the presence” if there was some doubt but the probability was greater than 50%.

3) “Possible about the presence” if there was reason to consider but the probability was less than 50%, and 4) “Definite about the absence” if there was no doubt about the absence.

‡ The categories “Definite and probable about the presence” were combined to one category and the categories “possible about the presence” and “definite about the absence” were also combined to one category.

The interobserver agreement was moderate to substantial for the signal intensity on T2 images; moderate for absence of epidural fat and flattening of the dural sac or the emerging root sheath; and slight for spinal canal stenosis (Table 5). When disc contour was dichotomized into “bulging” and “consideration of herniated disc” absolute agreement among the three observers was 95%.

**Table 5:** Interobserver agreement regarding characteristics of the impaired disc level.

	A vs B (n = 343)		A vs C (n = 329)		B vs C (n = 327)		All observers (n = 321)	
	%agreement	kappa	%agreement	kappa	%Agreement	kappa	%agreement	multiraterkappa
Disc contour ‡	95.9	*	98.2	*	95.1	*	95.0	*
Loss of disc height ∫	97.9	0.86	72.2	0.26	72.4	0.26	71.5	0.31
Signal intensity of nucleus pulposus on T2 images ¶	95.3	0.75	90.4	0.64	90.7	0.57	88.6	0.61
Type of vertebral endplate signal changes upper endplate∥	75.8	*	83.4	*	84.5	*	72.6	*
Type of vertebral endplate signal changes lower endplate∥	81.1	*	83.7	*	84.8	*	75.4	*
Spinal canal stenosis ∫	63.3	0.21	57.4	0.10	91.3	**	55.1	0.08
Absence of epidural fat adjacent to the dural sac or surrounding the nerve root sheath Ψ	74.0	0.52	74.1	0.54	73.6	0.54	61.7	0.50
Place of absence of epidural fat §	94.4	0.70	96.5	0.72	96.7	0.75	95.3	0.75
Impaired discs on other disc levels ∫	93.2	0.79	85.5	0.62	85.4	0.62	82.3	0.68
Nerve root thickness distal to the site of compression|--	93.5	***	93.5	***	97.5	***	92.1	0.40
Flattening of the ventrolateral angle of the dural sac or the emerging root sheath ∫	84.3	0.60	78.7	0.51	78.3	0.46	70.9	0.50

The number between brackets on the first row is the number of patients of which the observers suggested the same disc level as the symptomatic disc level. A en B represent the two neuroradiologists, while C represents the neurosurgeon.

‡ Categories were: bulging disc versus disc herniation.

∫ Categories were: yes versus no.

∥ Categories were: 1) Hypointensity 2) Normal 3) Hyperintensity.

∥ Categories were: 1) No vertebral endplate signal changes (VESC) 2) VESC type I 3) VESC type II.

4) VESC type III 5) Mixed VESC type I/II 6) Mixed VESC type II/III.

Ψ Categories were: 1) Yes, completely disappeared 2) Yes, partly disappeared 3) No disappearance.

§ Categories were: 1) Sub-articular zone 2) Foraminal zone 3) Extra-foraminal zone.

^|--^Categories were: 1) Normal 2) Thickened 3) Narrowed.

* Prevalence of findings too low (<10% of the reports) to calculate kappa values.

** Prevalence of spinal canal stenosis too low (<10% of the reports) to calculate kappa values.

*** Prevalence of thickened nerve roots too low (<10% of the reports) to calculate kappa values.

The interobserver agreement was excellent for side of the disc herniation and location on axial view; and moderate for location on sagittal view, size of disc herniation in relation to spinal canal and disc morphology (Table 6).

**Table 6:** Interobserver agreement regarding characteristics of the disc herniation.

	A vs B(n = 314)		A vs C(n = 313)		B vs C(n = 301)		All observers(n = 296)	
	%agreement	kappa	%agreement	kappa	%agreement	kappa	%agreement	kappa
Side of disc herniation|--	98.1	0.96	98.4	0.97	98.0	0.96	97.6	0.97
Location axial view ¶	94.2	0.88	95.5	0.90	96.7	0.93	95.6	0.92
Location sagittal view ∥	73.2	0.55	76.9	0.63	71.3	0.53	61.4	0.56
Size disc herniation in relation to spinal canal(4 categories) §	56.6	0.46	60.6	0.46	64.3	0.50	42.7	0.36
Size disc herniation in relation to spinal canal(2 categories) ‡	82.1	0.55	76.3	0.35	86.3	0.47	71.5	0.44
Protrusion versus extrusion	77.4	0.48	75.0	0.50	73.7	0.44	63.2	0.46

The number between brackets on the first row is the number of patients of which the observers suggested the presence of a disc herniation (on the same disc level). A en B represent the two neuroradiologists, while C represents the neurosurgeon.

^|--^Categories were: 1) Right 2) Left 3) Right and left.

¶ Categories were: 1) Central zone 2) Sub-articular zone 3) Foraminal zone 4) Extra-foraminal zone.

∥ Categories were: 1) Disc level 2) Folded upwards 3) Folded downwards.

§ Categories were: 1) Large ***stenosing: size >75%*** of the spinal canal ***2) Large: size 50–75%*** of the spinal canal 3) Average: size 25–50% of the spinal canal and 4) Small: size <25% of the spinal canal.

‡ The categories “large ***stenosing***” and “large” were combined to one category and the categories “average” and “small” were also combined to one category.

Intraobserver agreement regarding the probability of disc herniation and nerve root compression was higher among the neuroradiologists as compared to the neurosurgeon (Table 7). With use of a dichotomized scale absolute intraobserver agreement regarding nerve root compression ranged from 85 to 98%. Intraobserver agreement was substantial for spinal canal stenosis (kappa range 0.61–0.69); moderate to substantial for type of vertebral endplate signal changes (kappa range 0.52–0.74); fair to moderate for loss of disc height (kappa range 0.32–0.48) and flattening of the ventrolateral angle of the dural sac or the emerging root sheath (kappa range 0.30–0.52). Intraobserver agreement regarding the size and morphology of the herniated disc was fair to moderate (for size of the herniated disc kappa range 0.28–0.54, for morphology [extrusion versus protrusion] of the herniated disc kappa range 0.29–0.51).

**Table 7:** Intraobserver agreement among the three readers based on 40 MRI’s.

	Reader A		Reader B		Reader C	
	%agreement	kappa	%agreement	kappa	%agreement	kappa
Level that is assumed to cause the lumbosacralradicular syndrome ¶	97.5	*	90.0	*	87.5	*
Most affected nerve root	90.0	*	82.5	*	80.0	*
Probability of disc herniation (4 categories) ∫	95.0	*	92.5	*	70.0	*
Probability of disc herniation (2 categories) ‡	100.0	*	95.0	*	77.5	*
Probability of nerve root compression (4 categories) ∫	82.5	*	90.0	*	55.0	*
Probability of nerve root compression (2 categories) ‡	97.5	*	97.5	*	85.0	0.55
**Characteristics of the impaired disc level**						
Disc contour (consideration of disc herniation vs bulging) ∥	100.0	*	97.2	*	100.0	*
Loss of tdisc height§	84.6	0.42	77.8	0.32	74.3	0.48
Signal intensity of nucleus pulposus on T2 images Ψ	89.7	0.61	80.6	*	85.7	0.37
Type of vertebral endplate signal changes upper endplate|--	87.2	0.72	94.4	*	88.6	0.74
Type of vertebral endplate signal changes lower endplate|--	84.6	0.64	94.4	*	80.0	0.52
Spinal canal stenosis §	84.6	0.69	88.9	0.61	94.3	*
Absence of epidural fat adjacent to the dural sac or surrounding the nerve root sheath⊢	84.6	*	69.4	*	77.1	*
Place of absence of epidural fat adjacent to the dural sac or surrounding the nerve root sheath ζ	89.5	*	94.3	*	88.6	*
Impaired discs on other disc levels §	89.7	0.66	94.4	0.82	85.7	0.66
Nerve root thickness distal to the site of compression ∥–	82.1	*	97.2	*	88.6	*
Flattening of the ventrolateral angle of the duralsac or the emerging nerve root sheath §	79.5	0.51	83.3	0.52	71.4	0.30
**Characteristics the disc herniation**						
Side of disc herniation	100.0	1.00	94.3	0.89	100.0	1.00
Location axial view Ω	92.3	*	82.9	*	85.7	*
Location sagittal view Θ	87.2	0.81	82.9	0.71	71.4	0.56
Size disc herniation (4 categories) Ÿ	61.5	0.56	57.1	*	65.7	*
Size disc herniation in relation to spinal canal (2 categories) χ	76.9	0.54	74.3	0.28	85.7	0.37
Protrusion versus extrusion	76.9	0.51	82.9	*	68.6	0.29

Reader A en B represent the two neuroradiologists, while reader C represents the neurosurgeon.

* Since kappa values are afected by the prevalence of events, kappa values were only calculated for findings reported in more than 10% and less than 90% of all reports.

¶ The 5 categories were: 1) L2L3 2) L3L4 3) L4L5 4) L5S1 5) Not applicable, all disc levels have a normal disc contour: no disc extension beyond the normal margins of the intervertebral disc space.

∫ The 4 categories were: 1) Definite about the presence 2) Probable about the presence 3) Possible about the presence 4) Definite about the absence.

‡ The categories “Definite and probable about the presence” were combined and the categories “possible about the presence” and “definite about the absence” were combined to one category.

∥ Categories were: bulging disc versus disc herniation.

§ Categories were: yes versus no.

Ψ Categories were: 1) Hypointensity 2) Normal 3) Hyperintensity.

^|--^Categories were: 1) No vertebral endplate signal changes (VESC) 2) VESC type I 3) VESC type II.

4) VESC type III 5) Mixed VESC type I/II 6) Mixed VESC type II/III.

⊢ Categories were: 1) Yes, completely disappeared 2) Yes, partly disappeared 3) No disappearance.

ζ Categories were: 1) Sub-articular zone 2) Foraminal zone 3) Extra-foraminal zone.

∥– Categories were: 1) Normal 2) Thickened 3) Narrowed.

Ω Categories were: 1) Central zone 2) Sub-articular zone 3) Foraminal zone 4) Extra-foraminal zone.

Θ Categories were: 1) Disc level 2) Folded upwards 3) Folded downwards.

Ÿ Categories were: 1) Large ***stenosing: size >75%*** of the spinal canal ***2) Large: size 50–75%*** of the spinal canal 3) Average: size 25–50% of the spinal canal and 4) Small: size <25% of the spinal canal.

χ The categories “large ***stenosing***” and “large” were combined to one category and the categories “average” and “small” were also combined to one category.

## Discussion

This study showed excellent agreement between observers on the affected disc level (kappa range 0.81–0.86) and the nerve root (kappa range 0.86–0.89) that most likely caused sciatica in patients who were potential candidates for lumbar disc surgery based on clinical grounds. Among the three readers we found also substantial inter- and intra-observer agreement regarding the presence of disc herniation and nerve root compression when the four-point scale was dichotomized into “probability above 50%” and “probability lower than 50%”. Therefore, observer variation of MRI interpretation in potential candidates for lumbar disc surgery is satisfactory among spine experts regarding the characteristics most important in the decision for surgery. However, generally moderate agreement was found regarding the characteristics of the impaired disc level and the herniated disc. The moderate agreements may pose a problem when studying the relationships between specific imaging criteria and patient outcome.

Besides herniated discs, the direct evaluation of nerve roots and spinal canal by MRI has been considered an important asset to facilitate decision making in patients with leg and/or back pain [26], [27], [28]. Unfortunately, no universally accepted imaging criteria exist to define nerve root compression and lumbar spinal stenosis with MRI [6]. The interreader agreement regarding the presence of nerve root compression varies widely between studies. Cihangiroglu and co-authors found fair to substantial agreement (kappa = 0.30–0.63) between two neuroradiologists for classifying nerve root compression, which was dichotomized as absent or present, in 95 patients with low back or radicular pain [6]. Fair to moderate agreement was found for spinal canal stenosis. Van Rijn and co-authors found substantial agreement between two neuroradiologists when evaluating nerve root compression in 59 patients (kappa = 0.77) [29]. Their kappa is comparable with the agreement between the neuroradiologists in the present study (kappa = 0.80). Sorensen et al. found substantial agreement among two radiologists for classifying disc morphology of herniation (kappa = 0.68) in 50 low-field MRI scans [30]. Jarvik et al. evaluated imaging data from 34 patients with back pain [31]. Agreement between three radiologists for disc morphology was moderate to substantial with weighted kappa values of 0.50 to 0.75 across reader pairs. Interobserver agreement regarding the size and location of the disc herniation has been poorly investigated in previous studies. Characteristics of the disc level of the disc herniation (like signal intensity of the nucleus pulposus, loss of disc height, absence of epidural fat adjacent to the dural sac or surrounding the nerve root sheath, flattening of the dural sac or the emerging root sheath, and nerve root thickness distal to the site of compression) have also been poorly investigated in previous studies.

Our results indicate that the assessment of many variables is fairly subjective. However, it is crucial that radiologists and clinicians strive to reduce variability in interpretations as inconsistency in MRI interpretation may lead to alternative treatment options between clinicians and therefore may potentially impact the outcome of patient treatment [32], [33]. Previous studies reported that MRI findings play an important role in the decision for surgery [34], [35], [36]. Carlisle et al. observed that sciatica patients who underwent surgery had larger disc herniations and smaller spinal canals compared to nonoperative patients [34]. Cheng et al. observed that patients with either severe disc herniation or severe spinal stenosis were more likely to be classified as surgical candidates compared to those with mild to moderate findings [36]. Caragee and Kim also observed that patients who underwent surgery had larger disc herniations and smaller sizes of the remaing spinal canal compared to patients who underwent conservative treatment [35]. Besides that good reliability of imaging data in degenerative disc disease is important from a clinical point of view, it is also important for research purposes attempting to uncover the relationship between specific imaging characteristics and patient outcomes, which unfortunately remains controversial, with several studies showing a high prevalence of disc herniations in persons without any symptoms [37], [38]. To gain more insight in the relationship between MRI findings and patient outcomes, those interpreting the images must reliably assess the finding. One reason that a prediction model might lose its predictive power is the incorrect assessment of MRI findings, which causes the inputs in the prediction model to be faulty [39].

Within the literature, values of agreement on disc degeneration show a high variation depending on the variable investigated [40]. Although a few nomenclatures have been proposed, none has been widely recognized as authoritative or has been widely used in practice. This absence of consensus is greatly related to the multiple controversial aspects of disc abnormalities [41]. As a first step in the attempt to achieve better agreements between observers the language for image interpretation for degenerative disc disease has to be defined. Radiologists and clinicians should strive to define a nomenclature which has the best support among clinicians and radiologists. However, despite the adherence to predefined definitions in the present study, the MRI observers sill only reached moderate agreements regarding many characteristics of the disc level and the herniated disc, which indicate that definitions and the adherence to a well defined nomenclature only is probably not sufficient for reaching substantial to excellent agreements among observers. In addition to defining the language for image interpretation for degenerative disc disease, reading training might be an important next step [39], [42]. In support are the results of two reliability studies of The Spine Patient Outcomes Research Trial [3], [5]. In one of the two studies the reported agreement on disc morphology was only fair (kappa = 0.24) between the clinicians and radiologists [5]. In another study inter-reader reliability for disc morphology was excellent (kappa = 0.81) between 3 radiologists and 1 orthopedic surgeon [3]. The observation of a much better agreement in the second study might be explained by a better training of the MRI assessors as in that study the MRI assessors, before beginning the study, first evaluated a sample set of images with use of definitions and afterwards they met in person to review each image, enabling them to better streamline the way of interpreting the images.

When comparing kappa coefficients between studies caution should be exercised since there are other factors that can influence the magnitude of the coefficient, especially the number of categories and the prevalence of findings [43]. When the prevalence of findings is very low or high, kappa values also decline, even when the observed agreement remains unchanged [20], [23]. However, kappa remains the best available method to measure intra- and inter-observer agreement, in addition to that explained by chance [23].

We deliberately did not organize an extra meeting in which a sample subset of images was evaluated as the discussion during this meeting might have caused the observers to adjust their diagnostic imaging criteria. This may have led to an overestimation in the interpretation among the three readers compared to the situation as it existed before undertaking the meeting. During the meeting prior to the readings no images were evaluated, only a review of the questions and answers used in the case record forms to assure every reader understands their intended meaning when evaluating the images. If one does not undertake such a meeting this may pose problems when interpreting results as it may well be that a possible low observer agreement may not reflect true low agreement but agreement which arises due to the readers giving a different meaning to the questions or answers. We do not think such a meeting has a similar effect as evaluating together images before beginning the readings as then some observers may adjust their diagnostic criteria according to how other observers are evaluating the images during the meeting, with the consequence that one is not measuring the observer agreement as it existed before undertaking the meeting. Both procedures might lead to improving kappa coefficients, although more negative effects may arise when evaluating images together prior to the readings compared to only reviewing the questions and answers.

Our study has several limitations. An important limitation of the study is the number of observers, in particular the inclusion of only one non-radiologist, which limits the statistical power of the observer variation. Although all analyses were also conducted pairwise, the analyses in which all three observers are included should be carefully interpreted in light of the low statistical power. The inclusion of more observers having the same background, especially the inclusion of one more neurosurgeon in this study, would have strengthened the findings. The concordance found in this study may also have been overestimated, since one reading pair consisted of two neuroradiologists who had nearly the same observer experience and also worked together which may have led to an informal agreement in their diagnostic criteria [22]. Interestingly, however, the agreement between the neuroradiologists was sometimes lower compared to that of the reading pairs containing one of the two neuroradiologists and the neurosurgeon. The concordance might also have been overestimated since a great part of our study sample consisted of a relatively homogeneous study sample with well-defined inclusion criteria and known sciatica due to previous confirmed disc herniation by another observer. This might also explain why the observed agreement was lower among the patients who finally were not randomized [44]. However, as the presence of the disc herniations and nerve root compression was defined in different chance categories, the influence on the inter-reader reliability might have been limited. In addition, the use of standardized reporting forms with definitions and multiple choice categories allowed the assessments to be structured far more than possible in general clinical practice which also may have caused an overestimation [3]. Finally, usual reliable statistical packages (STATA, SAS) are only able to calculate unweighted kappa coefficients for multiple raters. However, unweighted kappa coefficients are inappropriate for ordinal scales since they treat all disagreements equally [43]. We encourage the development of statistical software that will solve this problem.

## Conclusions

The observer variation of MRI interpretation in potential candidates for lumbar disc surgery is satisfactory among spine experts with regard to clinically relevant parameters like most affected disc level and nerve root, probability of disc herniation and nerve root compression. However, in general considerable variation between the observers was found regarding specific characteristics of the symptomatic disc level and herniated disc. Therefore, it would be valuable to improve the reliability of image interpretation to subsequently increase our knowledge regarding the etiology, treatment and prevention of back pain and sciatica.
